# A Delphi survey of leadership attributes necessary for national nurse leaders’ participation in health policy development: an East African perspective

**DOI:** 10.1186/s12912-015-0063-0

**Published:** 2015-03-14

**Authors:** Nilufa Jivraj Shariff

**Affiliations:** Aga Khan University –School of Nursing and Midwifery, P.O. Box 39340, 00623 Nairobi, Kenya

**Keywords:** Leadership, Attributes, Nurse leaders, Participation

## Abstract

**Background:**

Nurses’ involvement in health policy development ensures that health services are: safe, effective, available and inexpensive. Nursing history reveals several legendary nurse leaders who have influenced policy and the course of nursing and health care. In the recent times there have been concerns regarding the availability of effective leaders physically, symbolically and functionally at clinical, organizational and national levels, who can effectively influence health policy. Exerting influence in the policy arena requires that nurse leaders acquire attributes that enable them to be effective in policy development activity.

This paper *reports part* of a larger study whose purpose included: *“build consensus on leadership attributes necessary for nurse leaders’ participation in health policy development in East Africa*”.

**Method:**

A Delphi survey was utilized and included the following criteria: expert panelists, three iterative rounds, qualitative and quantitative analysis, and building consensus. The study included purposively selected sample of national nurse leaders (expert panelists) from the three East African countries of Kenya, Tanzania and Uganda. The study was conducted in three iterative rounds. Seventy eight (78) expert panelists were invited to participate in the study and 37 (47%) participated in the first round of these; 24 (64.8%) participated in the second round and all invited in the third round 24 (100%) participated. Data collection was done using questionnaires and collected qualitative and quantitative data. Data analysis was done utilizing the principles of qualitative analysis in the first round and descriptive statistics in the second and third rounds.

**Results:**

The study achieved consensus on the essential leadership attributes for nurse leaders’ participation in health policy and include being able to: influence; communicate effectively; build relationships; feel empowered and demonstrate professional credibility.

**Conclusions:**

For nursing to participate in influencing the health policy and the health of the population, it will need to develop nurses with leadership attributes who are able to inspire change and influence the policy development process within the context where it exists. The leadership attributes identified in this study can be utilized to develop programmes geared to support nurses’ participation in health policy activity.

## Background

Nurses’ involvement in health policy development ensures that health care services are safe, effective, readily available and inexpensive [1]. The International Council of Nurses (ICN) [2] and World Health Organization (WHO) [3] recognize that nurses can make major contributions to health policy development toward promoting effective health systems. They call for nurses and midwives to be involved in policy decisions at all levels of the health systems. Furthermore, it is recognized that in order to achieve the Millennium Development Goals (MDGs), there is a need to provide countries with sufficient support to strengthen the nursing and midwifery workforce, leadership, and nursing involvement in policy making activities [4]. This paper reports part-finding from a broader study whose objective included to: *“build consensus on leadership attributes necessary for nurse leaders’ participation in health policy development in East Africa*”.

### International context of nursing leadership

Nursing history from the European and North American context reveals several renowned nurse leaders who have influenced the course of nursing and health care. For example, Florence Nightingale started the first nursing school and is recognized as the founder of modern nursing. Lillian Wald transformed public health nursing, Margaret Sanger led in the fight to offer women autonomy in reproductive rights, and Martha Franklin focused on concerns related to discrimination [5-9]. These distinguished nurses played exceptional roles in influencing policy development and reform [1]. They exhibited leadership attributes that included: being visionary; effective in communicating; networking; being change agents; exhibiting courage and taking risks; being creative; working collaboratively, exercising political astuteness by lobbying; and transforming situations [10-12].

In recent times, it has been noted that effective nursing leaders are not available physically, symbolically and functionally at clinical, organizational and national levels [13-16]. For example, a nursing study conducted in India reported that strong leadership is essential for the development of nursing, but reported a lack of visible nursing leadership [13]. Furthermore, nurses expressed frustration at the lack of leadership in relation to health policy activities by nurse administrators [17]. Campbell [18] found that nurse managers decried the lack of leadership and direction from senior administration. These studies indicate the need to support and build capacity of nursing leadership at senior positions to facilitate their ability to contribute to and influence policy decisions that address nurses concerns related to health care.

Effective leadership is critical to modern nursing’s potential to make a difference in health care and reform. To build the capacity of nurse leaders to effectively influence policy, it is important to understand the attributes that support them to function effectively at senior policy levels. Hennessy and Hicks [11] conducted a Delphi survey to examine the ideal attributes of chief nurses in Europe. The expert panelists ranked the sixteen most important attributes as follows: communication, promotion of nursing, strategic thinking, professional credibility, leadership, political astuteness, physical characteristics, personal qualities, team working, decency/integrity, innovation, good management, conflict resolution, information handling, research skills and decision-making/problem solving. Studies with nurse executives reported that communication, political advocacy skills, being knowledgeable and competent in nursing, being a team player, and possessing management skills, interpersonal skills, negotiation skills, being creative, working collaboratively, being visionary and having courage were important leadership attributes [19-21]. DiGaudio [22] established that nurses working in the policy arena viewed assertiveness and being proactive as positive traits for being involved in health policy activities.

Leadership attributes though must be considered contextually [23]. Lituchy, Ford, and Punnett [24] found that there were significant differences among groups with regards to attributes that effective leaders possessed, Ugandan participants valued being “honest and trustworthy”, Canadian and USA participants valued being “inspirational/charismatic”, while the Barbadians valued being “visionary”. Similarly, Leong & Fischer [25] found that leadership attributes differed across cultures and were contextual. Therefore, utilizing approaches and variables that are effective in one context might not be effective in another context.

### African context of nursing leadership

In the East African context, the Ministries of Health usually employ physicians as directors of medical services and the chief nurses’ report to them. The health care system generally accords more power, higher positions and remuneration to doctors. This has implications for nurses in relation to health policy where physicians and others are able to exert far greater influence on policy than nurses. This extends to matters that pertain to nursing as well. The number of nurse leaders in top national leadership positions within the national health systems is limited [26]. Amongst the nurse leaders who are in national leadership positions, a significant proportion of nursing leaders are excluded from policy development activities and therefore lack exposure to these processes [26]. Furthermore, the East African cultural and traditional systems are patriarchal where men are decision-makers at the household level; this is reflected in the overall health care system [27]. Subsequently, nurse Leaders’ face challenges related to: political skills, policy development skills, the status of women in a profession that is largely female, the image of nursing, lack of education, lack of nurturing and lack of supportive structures [28].

There is limited research literature pertaining to leadership and nursing leadership from the African context in general or from the East African context in particular. Notable exceptions are summarized here. In a study conducted by Jooste from South Africa found that nurse managers suggested that leaders should promote good interpersonal relationships through the attributes of being open, being inviting and empowering other nurses [28]. In contrast, Pillay [29] found that South African public sector nurse managers ranked control as the most important competency, followed by leadership, organization, and self-management. Participants evaluated themselves as being competent in self-management, planning, controlling, and leading. Koshal [30], explored the concept of servant leadership in various settings within the Kenyan context, and identified that role modeling, sacrificing for others, developing others, and services were regarded as the primary functions of leadership. Literature searches revealed limited information from the Global, African and particularly East African perspectives drawing attention to the *leadership attributes required for participation in health policy* activities by national nurse leader [31].

For nurses to effectively influence health policy and address concerns related to the health of the population, it is essential to assist nurses to develop leadership attributes that enable them to inspire change and influence policy development and reform. This paper *reports part* of a larger study that aimed to: explore the extent of nurse leaders’ participation in health policy development in East Africa; build consensus on factors that act as facilitators and barriers to nurse leaders’ participation in health policy development in East Africa; to develop an empowerment model that can enhance nurse leaders’ participation in health policy development and *build consensus on leadership attributes necessary for nurse leaders’ participation in health policy development in East Africa*, reported here.

## Methods

### Research design

A three round Delphi survey was applied and included the following: expert panelists, iterative rounds, statistical analysis, and consensus building. The method was utilized as it aimed to generate ideas from expert panelists own knowledge and experience in health policy development. The expert panelists were national nurse leaders from three East African countries. Furthermore, this approach can incorporate in the study *large numbers of participants* from geographically diverse locations and with the relevant expertise [32]. The study provided an opportunity to a panel of experts to communicate their opinions and knowledge anonymously, and to review their opinions, and to understand how their ideas align with others, and to change their opinions, if desired, after reviewing and reconsidering their own ideas in line with the group’s ideas [33,34]. Providing *anonymity* and confidentiality to the expert panelists, potentially prevented *dominance* by influential individuals and group pressure [35]. An important factor considered in this study was the positions that the panelists held (national nurse leaders); power differentials could have influenced the quality of the data had another method of data collection such as focus group interviews been utilized [36].

### Sampling

The study was conducted in the three East African countries of Kenya, Uganda and Tanzania. The sample consisted of expert panelists who were nurse leaders working in national or provincial leadership positions at the Ministry of Health (or equivalent), Nursing Councils, National Nurses Associations and Universities. A database of nurse leaders in senior leadership positions at national and provincial levels was developed to identify the expert panel members. Purposive sampling was used with the intent to include participants who were knowledgeable and had participated in health policy activity. The “closeness continuum” developed by Needham and de Loë (p.138) [37] was applied as a framework for including participants who would have the knowledge and experience to make a positive contribution to the study. As per the criteria proposed in the “closeness continuum”, nurse leaders with subjective expertise, mandated expertise and objective expertise were included in the study. A purposive sample of 78 expert panelists (nurse leaders) from East Africa was invited to participate in the study. Of these 37 expert panelists, 24 (64.8%) participated in the second round, and all 24 (100%) participated in the third round. The data collection process was conducted between September 2009 and May 2010.

### Round-one questionnaire development

The researcher developed the data collection tools. The first questionnaire included two sections: *Section 1 covered demographic data on the panelists with reference to* country represented, organization represented, number of years of experience in nursing, and number of years in current position. The demographic data helped to confirm that the sample was representative of nurse leaders as proposed in the sampling framework and possessed the critical characteristics relevant to achieving the aim of the study. *Section 2 aimed* to generate ideas from the expert panelist on leadership attributes necessary for participation in health policy development. Therefore, this objective was explored by asking the expert panelists an open-ended question, “what leadership attributes are essential to participate in health care policy development?”.

### Round-two questionnaire development

The aim of round 2 was to evaluate the level of consensus among the expert panelists on the leadership attributes identified from round 1, with a view to retain critical ideas for the next round. The participants were asked to evaluate the concepts presented to them in the light of their input in the first questionnaire and to review their views in relation to the views of others and to agree or disagree with these concepts which included: (1) influence; (2) communicate effectively; (3) build relationships; (4) feel empowered and (5) professional credibility (Table 1). The concepts identified in the first questionnaire informed the formulation and development of the second questionnaire. The second questionnaire contained closed ended questions and utilized a Likert scale that aimed at gathering information related to their level of agreement or disagreement (strongly agree; agree; undecided; disagree; strongly disagree) with the concepts presented [38].

**Table 1:** **Leadership attributes necessary for participation in health policy development (Round 1) (n = 37)**

		**Round 2 (n = 24)**			**Round 3 (n = 24)**		
		**PA**	**M**	**SD**	**PA**	**M**	**SD**
**Influence**	have *transformational* leadership attributes - being able to influence, being visionary and inspiring a shared vision	100%	1.25	0.53	100%	1.00	0.00
	have *negotiation skills* that generate win-win solutions	100%	1.21	0.42	100%	1.17	0.39
	be *politically astute* - able to lobby with policy makers and influence health policy of concern to nursing profession	100%	1.46	0.66	92%	1.5	0.78
**Communicate effectively**	have the ability to clearly *articulate* health issues of concern to nursing at policy development forums/arena	100%	1.21	0.42	100%	1.09	0.29
	be effective *communicators* who are able to articulate and disseminate health policy related issues – listening, speaking, writing	100%	1.21	0.42	96%	1.21	0.66
**Build relationships**	have effective *interpersonal* skills	100%	1.38	0.50	100%	1.27	0.46
	be able to cultivate *cordial* working relationships with colleagues and others within and outside the profession, in junior and senior positions	100%	1.42	0.50	100%	1.21	0.42
	be effective in *collaborating and cooperating* within and outside the profession	100%	1.33	0.48	100%	1.29	0.46
	be *team players*	100%	1.33	0.48	100%	1.09	0.29
	have *respect* for others	100%	1.48	0.67	100%	1.04	0.21
**Feel empowered**	be *proactive* and take initiative to formulate strategies of being involved at each stage of the policy development process	100%	1.33	0.48	100%	1.17	0.39
	have *personal confidence* through encouragement and a feeling of empowerment	100%	1.29	0.46	100%	1.77	0.38
	be *courageous* in articulating health issues of concern to nursing	100%	1.29	0.46	100%	1.13	0.34
	be *creative*	100%	1.26	0.54	100%	1.22	0.42
	be *motivated* to participate in health policy development	100%	1.46	0.59	96%	1.33	0.87
	be *assertive* in raising nursing concerns related to health care to policy makers	96%	1.38	0.77	100%	1.09	0.29
**Professional credibility**	have *management skills* – planning, organizing, supervising and evaluating	100%	1.25	0.53	100%	1.08	0.28
	be *knowledgeable and competent* in nursing	96%	1.58	0.97	100%	1.13	0.34
	have *critical thinking and problems solving* skills through nurse leaders education	96%	1.33	0.70	100%	1.17	0.39

### Round-three questionnaire development

The aim of round 3 was to evaluate the level of consensus among the expert panelists on the critical leadership attributes retained in round 2, and to retain the most important ideas. The participants were asked to re-evaluate the concepts presented to them in the light of their input in the first and second rounds and to review their views in relation to the views of others and to agree or disagree. As in the second round the questionnaire was condensed by merging similar concepts and close-ended questions were used. The same Likert scale utilized in round two was posted to the expert panelists who were expected to indicate their agreement or disagreement with the concepts presented.

### Pretesting questionnaires

Pre-testing of the questionnaires was conducted for all three rounds, utilizing a sample of nurse leaders who were excluded from the main study. The criteria for pre-testing the questionnaires were: length, clarity, language, congruence of content with study objectives, overall adequacy, and time taken to complete the questionnaire. No major modifications were recommended.

### Data collection process

A database with the current information pertaining to the contact information and positions of national nurse leaders was unavailable and was created. The database informed the researcher of the nurse leaders who would be best placed to participate and provide relevant information to meet the study purpose. All expert panellists included in the study were contacted and informed about the study. The participants were sent the 1^st^ questionnaire by post with a self-addressed stamped envelope and also emailed the same questionnaire. They were sent two reminders via emails. After six weeks no further reminders were sent and once the completed questionnaire was received from a participant, he/she was not sent any further reminders. The processes utilized in the second and third rounds were identical to the first round of data collection. In this study, expert panelist who participated in the first round were included in round 2; and those who responded to the second round included in the third round (Skulmoski, Hartman & Krahn [39]). Some studies are known to include the entire initial sample even those that did not respond. The rationale was that those who did not return questionnaires may not have responded because they were not sufficiently interested or thought they could not contribute towards the study. As indicated, from a purposive sample of 78 expert panelists (nurse leaders) in East Africa who were invited to participate, 37 (47.4%) did so in the first round, the 37 expert panelists were then invited to participate in the second round, 24 (64.8%) participated: all 24 (100%) participated in the third round. When the researcher was satisfied that no more questionnaires would be returned, the follow up exercises were terminated in all three rounds at approximately 6 weeks. The data collection process took six months.

### Data analysis

#### Qualitative analysis

The purpose of the first questionnaire was to generate ideas from the expert panelists. The first questionnaire generated unstructured, qualitative data. This data were transcribed verbatim into Word documents; the documents were thematically analyzed to identify reoccurring themes. The documents were examined for themes that were related and similar or dissimilar. The analysis of this phase was undertaken independently by the researcher and an assistant to ensure interrater reliability; notes were compared to validate the concepts that occurred. The themes identified were used to develop items utilized in the second and third round questionnaires.

#### Quantitative analysis

The purpose of the second and third rounds of the study was to build consensus on leadership attributes necessary for nurse leaders’ participation in health policy. The second and third rounds generated quantitative data that documented expert panelists’ evaluation and reevaluation of their ideas (consensus building) in line with group summaries and descriptive statistics. The quantitative data derived from the second and third rounds were analyzed utilizing the computer package Statistical Package for Social Scientists, version 15 (SPSS 15).

Descriptive statistics were used and included percentage agreement (PA), mean (M) and standard deviation (SD). Higher levels of agreement indicated higher levels of consensus related to the concepts. The statistical analyses were conducted to measure the level of agreement related to the concepts in the questionnaire.

The parameters set for the second round that indicated consensus and convergence of opinion towards agreement were: PA of ≥90%, M of <2 and SD of <2. The parameters set for the third round that indicated agreement were: PA of ≥70%, M of <2 and SD of <2. Therefore, a PA of <90% (2^nd^ round) or <70% (3^rd^ round), M of >2 and SD of >2 was considered divergence of opinion and lack of consensus. Consensus was built over three rounds, the first round generated ideas, the second and third rounds built consensus through the process by which the expert panelists evaluated and reevaluated their ideas.

#### Validity

Delphi surveys are primarily concerned with face and content validity [40]. *Face validity* was ensured through pre-testing the tool. Content validity was enhanced by firstly referring to the literature related to leadership attributes of nurse leaders. Secondly, the questionnaire was pre-tested with a representative sample of nurse leaders, to ensure that the concepts included in the study were actually related to leadership health policy development process. Thirdly, the purposive sample comprised experts who participated in health policy development processes.

### Ethical considerations

Ethical approval for the study was obtained from the three countries where the study was conducted. (National Council for Science and Technology of Kenya reference number - NCST/5/002/R/427; National Institute for Medical Research of Tanzania reference number - NIMR/HQ/R.8a/Vol.IX/904; and Uganda National Council for Science and Technology reference number - 698/07/1).

The participant’s right to autonomy was respected and informed consent attained by explaining the benefits, rights and risks involved in the research study in writing. Consent to participate was assumed by the return of the questionnaire. Confidentiality was maintained at all times throughout the study’s data collection phase. The questionnaires were accessible only to the researcher. The participants expressed their opinions anonymously; collected information was presented anonymously as group views, and data were presented in aggregate form, representing the collective views of the expert panel members [40,41].

## Results

### Demographic data

Demographic data were collected in the first round. A majority of the expert panelists who participated in this study were from Kenya where 30 (38%) were invited and 16 (43.2%) participated. From Tanzania, 34 (44%) were invited and 15 (40.6%) participated and from Uganda, 14 (18%) were invited and 6 (16.2%) participated. A majority of the expert panelists were above 40 years of age (n = 33; 89%) and were female (n = 23; 62%). Most of the expert panelists possessed a university degree (n = 26; 70%). Most panel members had considerable experience in the nursing profession: 32 (86%) had >15 years. Most had less experience in their current leadership position: 27 (73%) had ≤ 5 years of experience in this position.

### Leadership attributes necessary for nurse leaders’ participation in health policy development

Leadership attributes, identified by the expert panelists in the first round and achieved consensus in the subsequent rounds. These attributes included: (1) influence; (2) communicate effectively; (3) build relationships; (4) feel empowered and (5) professional credibility (see Table 1).

The attributes identified are shown in the first column of table one (1). The second and third rounds built consensus on these attributes and are shown in the second and third columns of the table. There was a high degree of (>92%) consensus achieved on all the leadership attributes, identified in the first round of the study, as necessary attributes for nurse leaders’ participation in health policy development (see Table 1).

## Discussion

The findings of the study indicate that certain leadership attributes are essential for nurse leaders’ ability to effectively participate in health policy development. These attributes are categorized under broad themes and include being able to: influence; communicate effectively; build relationships; feel empowered and demonstrate professional credibility. There was a high degree of consensus among the expert panelists on the essential leadership attributes in this respect (92% - 100%). It is speculated that the attributes that emerged in this study are attributes necessary for all professional nurses, nevertheless, the context, the depth and the complexity of the attribute may significantly differ in relation to participation in health policy development activity. This speculation is supported by Kelly, Wicker, and Gerkin’s [42] observation that there is a change in self-perceived leadership behaviors as leaders progress along the leadership path indicating that whilst the behaviours maybe the same there is enhanced growth and confidence in that attribute.

The current study’s findings concerning the essential attributes that support nurse leader participation in health policy development can be considered in relation to a study by Kunaviktikul, et al. [43] that explored the knowledge and involvement of clinical nurses and nurse leaders in health policy development. Bonding, unity, and leadership among nurses, as well as improving nurses’ political networks and improving their political skills were found to be necessary for enhanced involvement in health policy development activities. Although their study did not expand on specific leadership attributes, they identified barriers to participation in health policy activity included limited skills in public relations, which impacted the ability to explain and to promote what nurses do. Arguably, these skills are linked to ability to communicate effectively, build relationships, feel empowered, and demonstrate professional credibility - attributes identified in the current study. On the other hand, the East African social context is patriarchal and men are decision makers and women largely implement those decisions in the home and within the community, and nursing is a largely female dominated profession therefore this does pose barriers which need to be overcome through conscious nurturing of women for leadership.

Whilst literature that investigates leadership attributes necessary for participating in health policy activity is scarce, literature that explores leadership attributes of senior nurses provides relevant insights. The findings of the current study are consistent with leadership attributes identified for senior nurse leaders. Several studies [11,21,22,42-45] that have included samples of chief nurses, nurse executives and nurse leaders’ explored attributes and competencies of these nurses. Reported findings from those studies include attributes similar to those identified in the current study: communication, interpersonal skills, being visionary, negotiation skills, professional credibility and knowledge of nursing, political astuteness, team working, innovation, good management, collaborating and courage. This link between nursing leadership attributes and attributes necessary for participation in health policy development is suggested. Such a link is supported by findings of a study by McDaniels [46] whose study found that there is relationship between critical thinking and participating in policy activities. Moreover, there is evidence to suggest that political astuteness is linked to participating in legislative activities [46-48].

The current study did not divulge concepts related to empowering and enabling others. The findings of the current study suggest that nurse leaders need to be empowered and supported to participate in policy activity. Interestingly, the African region has long had the cultural concept of Ubuntu which demonstrates a spirit of humanism and caring for the individual and community has been long accepted. If East African nurse leaders’ intend to enhance and sustain their influence in the policy arena then developing an empowering and enabling environment might support sustained participation in the policy arena. The current study data neither disclosed attributes related to ethics such as integrity and valuing diversity, nor concepts related to being an evidence based leader or policy maker [22]. Nurse leaders’ might have to work towards fostering attributes that include valuing diversity and inclusiveness within nursing leadership, for sustained participation in policy and decision making.

### Limitations of the study

There are several limitations to this study. Firstly, the study was conducted in the three East African countries of Kenya, Tanzania and Uganda. Therefore, the findings are only applicable to the countries where the study was conducted. Purposive sampling was applied to this study and the sample was selected as per the researcher’s knowledge of the contribution that the expert panelists could make to the study. This may have resulted in some relevant nurse leaders and their knowledgeable being excluded. A disadvantage of Delphi approach cited in the literature is related to a clear definition of consensus. The literature suggests that 51% to 70% agreement represents consensus [40,41]. The consensus level of agreement set for this study was high, at 90% for the second round and 70% for the third round. This might have led to elimination of some important issues.

### Recommendations

The findings of the study indicate that there are essential leadership attributes that nurse leaders require to effectively participate in health policy development. Nursing leaders, educators and researchers need to facilitate nurses in gaining competence pertaining to leadership in the health policy domain. This would create a larger pool of candidates who may be interested in participation in health policy activities. That in turn might serve to further nurses’ interests on issues of concern to nurses in the health policy arena. This study is a beginning in this field from the East African context and it is recommended that additional research be conducted to advance and expand on the knowledge gained in this study. Furthermore, replication of the study with other samples will contribute in this field of nursing. As a follow up of this study further research is intended to explore how the expert panelists, who were able to influence health policy, were able to do so from within the context of East Africa.

## Conclusion

The study is unique to the African context and the particular focus on the Kenya, Uganda and Tanzania, where little is known about the leadership attributes necessary for nurse leaders’ participation in health policy activities. The study utilized the Delphi approach to build consensus on the leadership attributes necessary for participation in health policy development. The findings identify essential leadership attributes that include being able to: influence; communicate effectively; build relationships; feel empowered and demonstrate professional credibility. These attributes can be learned. For nursing to participate in influencing the health policy and the health of the population, it will important, arguably essential, to develop nurses with leadership attributes that enable them to inspire change and influence the policy development process within their context. The leadership attributes identified in this study can be utilized to develop programmes geared to support nurses’ participation in health policy activity. Effective participation in health policy development requires nurse leaders who are able to exert influence and are visionary and inspiring. The health policy development arena is highly political with stakeholders exerting pressure towards achieving their own ambitions with regards to health policy development. Political astuteness enhances nurse leaders’ ability to apply pressure on the health policy development process, by being knowledgeable about health and nursing issues of concern to nurses, contacting and communicating effectively with policy makers, lobbying, building relationships, and willing to testify in policy forums.
